# Emergency Department Use across 88 Small Areas after Affordable Care Act Implementation in Illinois

**DOI:** 10.5811/westjem.2017.5.34007

**Published:** 2017-07-17

**Authors:** Joe Feinglass, Andrew J. Cooper, Kelsey Rydland, Emilie S. Powell, Megan McHugh, Raymond Kang, Scott M. Dresden

**Affiliations:** * Northwestern University Feinberg School of Medicine, Division of General Internal Medicine and Geriatrics, Chicago, Illinois; † Northwestern University, Northwestern University Library, Evanston, Illinois; ‡ Northwestern University Feinberg School of Medicine, Department of Emergency Medicine, Chicago, Illinois; § Northwestern University Feinberg School of Medicine, Center for Healthcare Studies, Chicago, Illinois

## Abstract

**Introduction:**

This study analyzes changes in hospital emergency department (ED) visit rates before and after the 2014 Affordable Care Act (ACA) insurance expansions in Illinois. We compare the association between population insurance status change and ED visit rate change between a 24-month (2012–2013) pre-ACA period and a 24-month post-ACA (2014–2015) period across 88 socioeconomically diverse areas of Illinois.

**Methods:**

We used annual American Community Survey estimates for 2012–2015 to obtain insurance status changes for uninsured, private, Medicaid, and Medicare (disability) populations of 88 Illinois Public Use Micro Areas (PUMAs), areas with a mean of about 90,000 age 18–64 residents. Over 12 million ED visits to 201 non-federal Illinois hospitals were used to calculate visit rates by residents of each PUMA, using population-based mapping weights to allocate visits from zip codes to PUMAs. We then estimated n=88 correlations between population insurance-status changes and changes in ED visit rates per 1,000 residents comparing the two years before and after ACA implementation.

**Results:**

The baseline PUMA uninsurance rate ranged from 6.7% to 41.1% and there was 4.6-fold variation in baseline PUMA ED visit rates. The top quartile of PUMAs had >21,000 reductions in uninsured residents; 16 PUMAs had at least a 15,000 person increase in Medicaid enrollment. Compared to 2012–2013, 2014–2015 average monthly ED visits by the uninsured dropped 42%, but increased 42% for Medicaid and 10% for the privately insured. Areas with the largest increases in Medicaid enrollment experienced the largest growth in ED use; change in Medicaid enrollment was the only significant correlate of area change in total ED visits and explained a third of variation across the 88 PUMAs.

**Conclusion:**

ACA implementation in Illinois accelerated existing trends towards greater use of hospital ED care. It remains to be seen whether providing better access to primary and preventive care to the formerly uninsured will reduce ED use over time, or whether ACA insurance expansion is a part of continued, long-term growth. Monitoring ED use at the local level is critical to the success of new home- and community-based care coordination initiatives.

## INTRODUCTION

The 2014 Affordable Care Act (ACA) insurance expansions were designed to increase access to care and potentially lower hospital costs associated with undiagnosed, unaddressed health care problems that often result in visits to a hospital emergency department (ED). By increasing access to primary care, it was hoped that ACA insurance expansions might, at least over time, reduce ambulatory care sensitive hospital use.^1^ The ACA’s 2010 private insurance expansion for young adults has been associated with reduced ED use,^2^ and some studies have found no increases in ED use after the first year of the ACA.^3^,^4^ Other studies have found that ED use for young adults with non-discretionary conditions increased,^5^ and previous state-level insurance expansions and even county-level access to care programs that include the older uninsured population have often resulted in significant increases in ED use.^6^–^12^ Gaining health insurance is associated with significant financial, mental health, access to care, self-reported health status and mortality gains.^13^–^19^ Although reducing ambulatory care sensitive ED use was one aim of ACA insurance expansions, the literature on prior insurance and access to care expansions generally predicts higher ED use when newly insured patients pursue a backlog of previously unaddressed health issues.^20^

A recent statewide analysis of ACA effects on Illinois ED visits for the 18- to 64-year-old population found an approximate 5% increase in ED visits above and beyond pre-existing time series trends in the two years after full implementation of the ACA.^21^,^22^ This follow-up study presents findings on how the 2014 ACA insurance expansions affected ED visit rates cross 88 Public Use Micro Areas (PUMAs) in Illinois. PUMAs, with an average of about 90,000 age 18–64 residents, reflect a remarkable range of pre-existing ED use rates across diverse urban, suburban and rural community areas of Illinois. We used the 88 Illinois PUMAs, the lowest level of aggregation for U.S. Census data on insurance status, to correlate changes in population-level insurance status with concurrent changes in area ED use at 201 Illinois hospitals between 2012 and 2015. We present methods that are replicable for any state with publicly available, zip-coded ED hospital claims data.

We first analyzed census estimates of area changes in insurance status, rarely analyzed at the small-area level. We then describe correlations with concurrent changes in ED use rates across a wide variety of urban, suburban and rural community areas in Illinois between 2012 and 2015. While further documenting the association between insurance expansion and ED use in Illinois, findings provide insight into the striking variation in ED use across socioeconomically diverse communities. The methods used here for Illinois here can be replicated for any state by mapping state zip-coded hospital ED visit claims to PUMA-level census data. While many studies are appropriately focused on inter-state comparisons, the advent of ACA insurance expansions in 2014 provides a unique lens on small-area dynamics in hospital emergency care utilization.

Population Health Research Capsule

What do we already know about this issue?

Like previous insurance expansions, implementation of the Affordable Care Act (ACA) in 2014 led to an increase in emergency department (ED) visits for the 18- to 64-year-old population in Illinois.

What was the research question?

This study examines large variations in ED visit rate changes across 88 socioeconomically diverse areas of Illinois.

What was the major finding of the study?

Areas with the largest increases in Medicaid enrollment experienced the largest growth in ED use.

How does this improve population health?

Better access to emergent care for the previously uninsured may be one reason why state insurance expansions have been found to improve population mortality rates.

## METHODS

This study analyzes PUMA-level annual American Community Survey (ACS) insurance estimates and PUMA-level changes in ED visits, including hospitalization through the ED, across 88 Public Use Micro Areas (PUMAs) in Illinois. The effect of ACA insurance expansion was measured by the change in average monthly ED visit rates per 1,000 PUMA residents between the 24-month pre-ACA (2012–2013) period and the 24-month post-ACA (2014–2015) period. We analyzed the correlation between PUMA-level change in population insurance status and concurrent change in PUMA-level ED visit rates.

### American Community Survey Population Estimates of Area Insurance Status

PUMAs are the lowest level of aggregation for census population level insurance status estimates from the annual American Community Survey (ACS) for the years 2012–2015.^23^ Annual ACS insurance population estimates are based on approximately 100,000 interviews in Illinois each year and have <2% statewide margin of error for insurance status estimates of the 18–64 population. PUMAs had an average of just over 90,000 residents age 18–64. The number of residents age 18–64 who reported being uninsured, or being primarily covered by Medicaid, Medicare (disability), private insurance or other coverage (e.g. VA or TRICARE) were derived for each PUMA in Illinois from 2012–2015 annual ACS estimates.^23^ Because some residents reported multiple insurance coverage, combined estimates of all insurance categories resulted in a small over-count. We also obtained cross-sectional estimates of the percent of area residents below poverty level the number who were disabled and/or non-citizens and the area median household income, from 2010–2014 five-year PUMA-level ACS estimates.

### Hospital Administrative Data on ED Visits

Hospital administrative data from 201 non-federal Illinois hospital EDs were obtained from the Illinois Hospital Association Comparative Health Care and Hospital Data Reporting Services (COMPdata) database. We analyzed records for patient demographics, hospital ED visits, and admissions through the ED for all patients age 18–64 with Illinois zip codes from January 2012 through December 2015, a 48-month study period. Patient zip codes were used to impute median household income using census estimates from zip code tabulation areas (ZCTA) from the five-year (2009–2013) ACS. Low-income zip codes were defined as having median household income below $35,000. All data were de-identified and the study was ruled exempt by the Northwestern University Institutional Review Board.

### Mapping Zip Codes to PUMAs and Calculation of Monthly ED Visit Rates

We used the geographic cross-mapping utility of the University of Missouri Census Data Center to identify census ZCTAs whose boundaries overlapped more than one Illinois PUMA.^24^ We weighted hospital ED data at the ZCTA level so that ED visits for patients living in boundary-crossing ZCTAs could be apportioned to PUMAs based on 2012 estimates of the proportion of each ZCTA’s residents living in each PUMA. Thus, if one ZCTA had 80% of its population living in one PUMA and 20% living in a second PUMA, 80% of ED visits of residents of that ZCTA were attributed to the first PUMA and 20% to the second. Rates per 1,000 PUMA residents were then calculated from monthly visit data numerators and PUMA age 18–64 annual total and insurance status population denominators.

### Statistical Analysis

ED visit rates per 1,000 residents for each of the 88 Illinois PUMAs were aggregated monthly and for each separate insurance primary payer over the 48-month study period for each PUMA’s 18–64 population. We compared average monthly ED visit rates during the 24-month, pre-ACA period to the same rates observed in the 24-month post-ACA period. For each PUMA, we calculated the mean ED visit rate difference between time periods as a difference in absolute numbers, the absolute rate difference per 1,000 residents, and as a percent change difference from the baseline rate. Pearson correlation coefficients tested the significance of bivariate associations between ACA-related PUMA population change in each insurance status and change in PUMA residents’ monthly ED use rates. Using linear regression, we tested the effects of simultaneous change in insurance (Medicare disability, Medicaid, uninsured, private and other/unknown) across all categories, and controlled for fixed estimates of PUMA sociodemographic characteristics (percent of area residents below poverty level who were disabled, who were non-citizens, and area median household income). Analyses were performed with SPSS Version 22, Chicago, Illinois and SAS Version 9.4, Cary, North Carolina.

## RESULTS

### Changes in Insurance Status Across Illinois

In the pre-ACA period, approximately 70% of Illinois residents age 18–64 reported private insurance coverage, 18% reported being uninsured and just over 10% reported Medicaid coverage. However, there was wide variation across PUMAs. Only 31.5% of residents from Chicago’s West Side Lawndale, Humboldt Park and Garfield Park PUMA reported private coverage, and 41.1% reported being uninsured. This compared to 90.3% reporting private coverage and only 6.7% reporting being uninsured in Western Kane County in exurban Chicago. Only 2.0% of residents in the Near North, Loop and Near South Side in downtown Chicago reported Medicaid coverage as compared to 29.9% of residents of the Chicago Lawn, Englewood and Grand Crossing neighborhoods on the South Side of Chicago.

**Figure 1:**
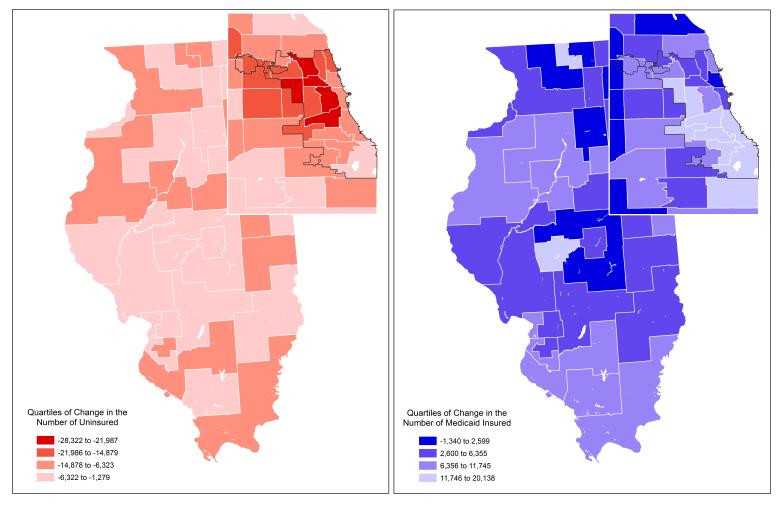
Quartiles of change in annual American community survey estimates of residents age 18–64 (A) uninsured, or (B) with Medicaid coverage for 88 Illinois public use micro areas.

Averaged statewide-census insurance status estimates of changes between 2012–2013 and 2014–2015 were modest, with a +2.8% increase in private and a 3.3% change in Medicaid coverage, a 4.7% decline in uninsurance, and virtually no change (0.1%) in Medicare disability coverage. However, these average change data mask much more significant variation in insurance transitions across PUMAs, as shown in Figure 1 for the uninsured and for residents covered by Medicaid. This figure maps ACS estimates by quartiles of the absolute number of PUMA residents with insurance status changes between the pre- and post-ACA periods. The darkest red PUMAs in Figure 1 had the largest absolute declines in the number of uninsured residents; conversely, the lightest blue areas had the largest absolute increases in Medicaid enrollment between periods. Changes in insurance coverage ranged from less than a 1% decline in uninsurance in suburban Plainfield and Lockport townships in suburban Will County to a 15.8% decline in Aurora. Changes in Medicaid enrollment ranged between a 1% decline in affluent areas of suburban Lake and Will counties to a 10.6% increase in the South Side Chicago neighborhoods of Auburn-Gresham, Roseland, Chatham, Avalon Park and Burnside. Six PUMAs had greater than 20,000-person declines in uninsured residents, and another 10 PUMAs had over 15,000-person declines. Conversely, six PUMAs had more than a 15,000 resident increase in Medicaid coverage and nine others had an over 10,000 resident increase. Figure 1B provides a PUMA map of Illinois and Chicago area changes in private insurance coverage; the darkest purple areas had a >5,000 resident gain in privately insured residents.

### Changes in Emergency Department Visit Rates

**Figure 2:**
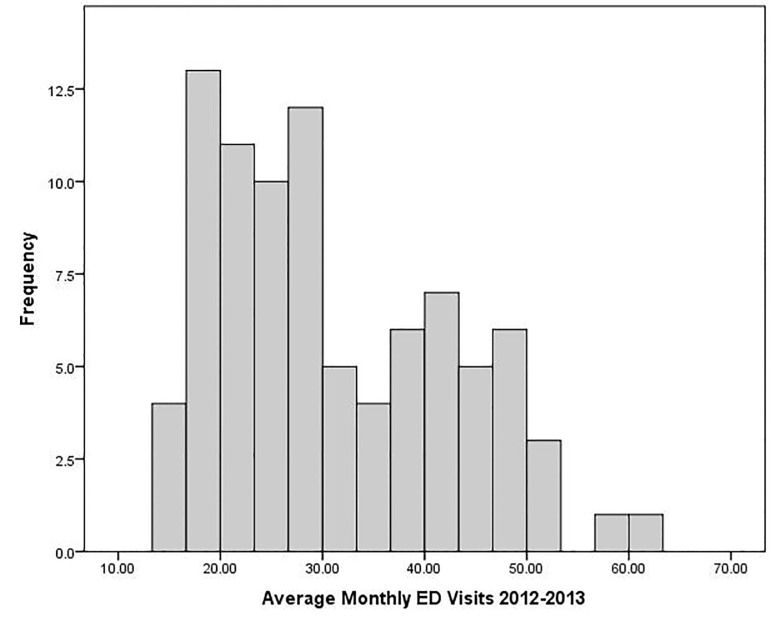
Average 2012–13 monthly emergency department (ED) visits per 1,000 residents age 18–64 in 88 Illinois public use micro areas.

**Figure 3:**
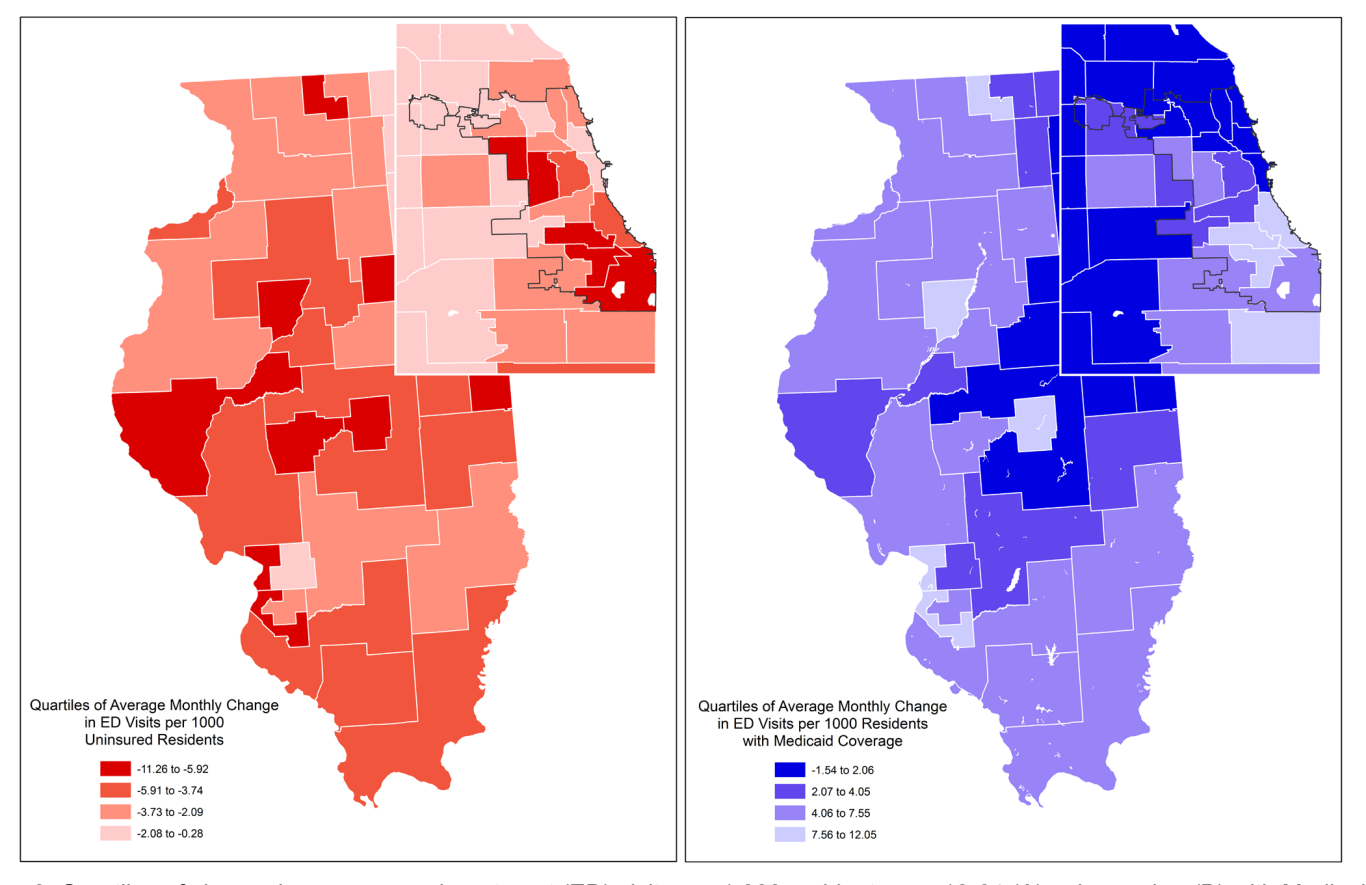
Quartiles of change in emergency department (ED) visits per 1,000 residents age 18–64 (A) uninsured or (B) with Medicaid coverage across 88 Illinois public use micro areas.

There were over 12.28 million ED visits for Illinois residents age 18–64 over the 48-month study period. The proportion of visits that resulted in hospitalizations through the ED actually decreased from a mean of 11.75% of visits pre-ACA to 11.2% post-ACA; mean monthly ED admissions increased very slightly from 29,031 in 2012 to 29,503 in 2015. Pre-ACA average monthly ED visit rates per 1,000 (Figure 2) ranged from 13.5 in the Lake View and Lincoln Park neighborhoods on Chicago’s North Side to 62.3 in the Lawndale, Humboldt Park and Garfield Park neighborhoods on Chicago’s West Side. Across all areas, for 18–64 year old residents, average monthly ED visit rates increased from 31.2 per 1,000 from 2012–2013 to 36.6 per 1,000 in 2014–2015, while ED admission rates per 1,000 residents actually decreased slightly. Figure 3 maps quartiles of change in average monthly ED visit rates for uninsured and Medicaid patients between 2012–2013 and 2014–2015. The darkest red PUMAs had *declines* in uninsured visits of >5.9 per 1,000 while the lightest blue PUMAs had *increases* in Medicaid visits of >7.6 per 1,000.The greatest overall post-ACA decline in average monthly visit rates was −3.0 per 1,000 in downstate Montgomery, Bond, Clinton, Fayette and Effingham counties, while the largest overall increase was 10.8 per 1,000 in the Lawn, Englewood, and Grand Crossing neighborhoods of Chicago’s South Side. Statewide, average monthly visit rates for the uninsured fell by 3.1 per 1,000 in 2014–2015 while average monthly Medicaid visits increased by 3.6 per 1,000. The largest decline in average monthly visits for uninsured residents (−11.3) was in Adams, Pike, Brown, Schuyler and Mason counties in western Illinois and the largest increase in monthly Medicaid visits was in Rockford and surrounding Winnebago County (+12.0). Figure 3B provides the same map for changes in all ED visits for Illinois- and Chicago-area PUMAs, with the lightest purple areas reflecting overall monthly ED visit increases of over 4.7 visits per 1,000.

### Associations between Changes in Population Insurance Status and Changes in Visit Rates

**Figure 4:**
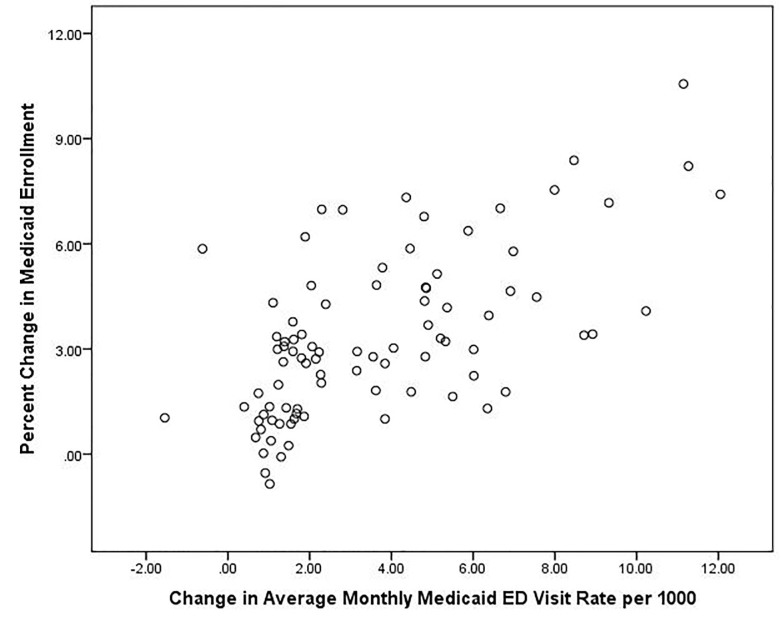
Correlation between change in Medicaid enrollment and change in average monthly Medicaid emergency department (ED) visits before (2013–14) and after (2014–15) Affordable Care Act insurance expansion. r=0.63, p<0.001.

Average monthly Medicaid ED visits rose from 8.6 per 1,000 (SD=5.2) to 12.2 per 1,000 (SD=7.6), a 41% increase over the pre-ACA baseline, and had by far the highest correlation (r=0.63, p<0.001) between changes in coverage and changes in ED visit rates (Figure 4). The table displays PUMA-level insurance-specific changes in average monthly ED visit rates in the first column, showing a larger than offsetting increase in the Medicaid visit rate as compared to the decrease in uninsured visits. The second column of the table presents bivariate correlations between the percent change in each insurance population and pre-post ACA change in ED average monthly visit rates for all PUMA residents. Changes in ED visit rates for the uninsured and residents covered by Medicare disability were not significantly correlated with change in their coverage, while change in the ED visit rate by the privately insured (+1.1, SD=1.7) was modestly but non-significantly correlated with change in PUMA private insurance coverage (p=0.06).

The final columns of the table present linear regression results for the association of all insurance- population changes simultaneously with change in total average monthly ED visit rates. PUMA cross-sectional census characteristics were tested in an initial model of change in total ED visit rates, before entering what were expected to be highly correlated insurance status changes. PUMA percent poverty (p=0.08) and percent disabled (p=0.05) were modestly correlated with ED visit rate changes, but after entering insurance status changes, all cross-sectional census characteristics became non-significant (and had virtually no effect on insurance status coefficients, data not shown). Only change in Medicaid coverage was significantly associated (p<0.001) with change in overall average monthly ED visit rates in the multiple regression model. Change in insurance status explained about a third of the variance in overall ED visit rate change across PUMAs.

## DISCUSSION

### ACA Insurance Expansion and the Long-Term Increase in ED Use

The regional differences we describe within Illinois provide insight into long-debated policy issues about the role of hospital emergency care in the U.S. healthcare system.^25^ ED use among non-elderly adults, especially for “primary care treatable” or lower acuity conditions, was already higher and has been growing most quickly for Medicaid patients.^26^–^28^ NHIS data for the 18–64 population indicate that in 2014, 35.2% of respondents with Medicaid, 14.3% with private coverage and 16.6% who were uninsured reported visiting the ED one or more times that year.^29^ This disparity reflects both differences in health status and enduring barriers to timely office- or home-based acute care for low-income patients, as well as numerous care-coordination failures for patients with chronic or end-of-life illness.^30^–^32^

Like other states, Illinois has seen the growth of managed care providers and patient-centered medical home efforts, which seek to reduce ED use.^33^ There has also been a rapid increase in non-hospital urgent and immediate care centers, although these disproportionally serve higher-income areas and do not seem to affect hospital ED rates.^34^,^35^ Yet in Illinois and other states, lower-income, uninsured, underinsured and Medicaid patients, including many individuals with unaddressed psychosocial dimensions to multimorbidity, continue to experience major obstacles to accessing timely primary and behavioral healthcare.^36^

In the U.S. primary care is often inaccessible during work-week evenings and weekends,^37^ and a greater proportion of ED care is being devoted to managing high-acuity visits by patients with undifferentiated complaints, in part because time-pressured primary care providers are increasingly sending medically and socially complex patients to the ED for diagnosis and treatment.^38^,^39^ Reducing financial barriers to ED care with ACA insurance expansion in Illinois, which has a relatively fixed supply of ED beds, appears to have exacerbated existing trends towards increasing ED visit rates.

A recent study of claims data from 126 investor-owned hospitals also found a significant post-ACA increase in ED use in Medicaid expansion states, as opposed to nonexpansion states.^40^ Of note, this study found that Medicaid patients in expansion states experienced a 6.2% decreased travel time to hospitals, almost certainly concentrated among the newly insured. This decrease in travel time was pronounced for Medicaid patients with more severe, non-discretionary conditions, two thirds of whom do not arrive by ambulance. Increased ED use may thus be a potentially important factor underlying the observed mortality reductions that accompany insurance expansions.^16^

### Small Area Variation in ED Use

This study provides two years of post-ACA data to evaluate change in ED use in Illinois. Our PUMA-level analysis shows that changes in ED use related to the ACA in Illinois are rooted in wide differences in local and regional ED use rates, with the underlying variation in area ED visit rates poorly understood. There have been few recent studies of community differences in ED use, and fewer longitudinal studies of area-level changes in ED rates over time.^41^

The variation in ED use across Illinois PUMAs reflects, in part, the well-studied variation in small-area medical and surgical hospitalization rates.^21^ Explanations of variation in small-area hospital use remain divided about the extent to which use rates reflect supplier-induced demand for hospital care, as opposed to area differences in illness^42^ or higher healthcare use by low-income residents within hospital market areas.^43^

Writing over a decade ago about a 20-fold variation in ED use within Robert Wood Johnson Community Tracking Study communities, Cunningham et al. found correlations between ED use and area primary care physician- and hospital-supply characteristics, but little correlation between ED use and the number of uninsured area residents.^41^ Variation in ED admission rates across small areas also reflects the impact of local medical norms on clinical policy.^44^–^46^ Recently**,** Pines et al. found that county-level differences in admissions through the ED were negatively correlated with primary care physician supply,^44^ but the role of primary care physician “density” remains controversial and differs between geographic units.^46^

### Implications for Delivery System Reform

Administrative and copayment financial sanctions to reduce “non-acute” ED care have largely failed and may be unethical.^47^,^48^ Recent population health incentives have highlighted ED-based care coordination interventions targeted to patients who receive the most fragmented care and have the highest likelihood of hospital admission through the ED.^49^ These efforts will need to directly address social services and social determinants of health as they manifest in highly local settings.^50^ It is worth considering how shifting to a delivery system based on home- and community-care coordination may change ED use and ED practice in coming years, and how such changes can be tailored to particular community values.^51^–^53^ For Illinois communities, this report serves as a benchmark for future initiatives seeking to reduce ED use and makes clear which areas are most in need of care coordination investment and infrastructure.

## LIMITATIONS

There are several potentially significant limitations to this study. ACA Medicaid expansion began in Illinois in January 2013 with a federal waiver for early ACA Medicaid enrollment in Cook County. By mid-2014, over 100,000 people in the Chicago area had become newly enrolled, and some new Chicago-area Medicaid enrollees were receiving services in 2013. While this biases ED use estimates upwards for the pre-ACA period analyzed here, it makes the January,2014 ACA cut off more appropriate since by then newly registered patients were already obtaining services from new county Medicaid-managed care programs.

Illinois hospital claims data, which reflect multiple visits by the same patient, will not be commensurate with self-reported National Health Interview Study (NHIS) data on the number of respondents reporting they visited the ED during the previous year.^4^ One national 2013–2014 NHIS study shows a 5% decrease in Medicaid ED visits, a 3.3% decline in uninsured ED visits and a 3.9% increase in privately insured ED visits,^54^ and based on trends over 2010–2014, residents of Medicaid expansion states had modestly higher post-ACA ED visits and overall hospitalization rates.^4^

Repeated use of the ED represents a substantial proportion of all visits and especially Medicaid visits.^55^,^56^ Our visit data are not patient-identified and we cannot speculate on ACA effects on frequent ED use, nor distinguish use by patients newly enrolled in Medicaid. Nor do we have claims or census data, which differentiate employer-sponsored privately insured vs. self-purchased policies. We excluded ED visits for non-Illinois residents, which represent about 2.5% of all visits to Illinois hospitals; also excluded were a smaller number of ED visits by Illinois residents to hospitals in other states.

## CONCLUSION

We found that areas with the greatest increases in Medicaid enrollment had the highest overall ED visit rate increases. Our findings on ED use in Illinois support the hypothesis that because insured patients gain the financial security to use the ED for previously unaddressed health issues, there will be an expected ED use spike after access expansions remove financial barriers to care.^20^ It remains to be seen whether eventually providing better access to primary and preventive care to the formerly uninsured will reduce ED use over time, or whether ACA insurance expansion is just a small part of continued long-term growth.

## Figures and Tables

**Table:** Correlations between changes in insurance coverage for residents age 18–64 and changes in average monthly emergency department (ED) visits between 2012–13 and 2014–15 across 88 Public use micro areas in Illinois.

	Change in average monthly ED Visit rates per 1000 residents between 2012–13 and 2014–15 (range)	Bivariate correlations with percent changes in area insurance coverage		Linear regression of percent changes in total monthly ED visits(1)		
			
		Pearson correlation coefficient	p value	b	SE	p value
Uninsured	−42 (−68 to −10)	−0.12	0.26	0.09	0.09	0.32
Medicaid	+42 (+76 to −19)	0.63	<0.001	0.63	0.10	<0.001
Private insurance	+10 (+83 to −14)	0.20	0.06	0.008	0.01	0.91
Medicare (disability)	−5 (+30 to −22)	0.13	0.24	0.37	0.46	0.93

*ED*,emergency department; *SE*, standard error.

(1) R^2^=0.33 Change in other or unknown insurance is the reference category.
